# HPV prevalence and type distribution in women with or without cervical lesions in the Northeast region of Romania

**DOI:** 10.1186/1743-422X-8-558

**Published:** 2011-12-22

**Authors:** Ramona Gabriela Ursu, Mircea Onofriescu, Dragoş Nemescu, Luminiţa-Smaranda Iancu

**Affiliations:** 1 Microbiology Department, "Grigore T. Popa" University of Medicine and Pharmacy, Universitatii Str. NO 15, Iaşi 700115, Romania; 2 Gynecology Department, "Grigore T. Popa" University of Medicine and Pharmacy, Iaşi, Universitatii Str. NO 15, Iaşi 700115, Romania

**Keywords:** HPV, Screening, Cytology, Prevalence, Persistence, Vaccination

## Abstract

**Background:**

Cervical cancer is a major public health problem worldwide. While Romania has the highest incidence of cervical cancer in Europe, the prevalence of HPV has not been evaluated. We report the first data on HPV prevalence and type distribution in Northeast Romania.

**Methods:**

HPV prevalence and genotype distribution was investigated in 514 consecutively women with or without cervical lesions in Northeast Romania. Genotyping was performed with Linear Array Genotyping/Roche kit.

**Results:**

In our study group, 192/514 (37.4%) patients were positive for HPV (infected with single and with multiple HPV types). Most frequent types were: 16 (10.5%), 53 (5.44%), 51 (5.05%), 52 (4.08%) 18 (2.91%) and 31 (2.73%).

**Conclusions:**

Infection with high risk types of HPV is common in Northeast Romania. Enhanced and systematic screening for cervical cancer is needed. Our results call for the implementation of a National HPV vaccine program in Romania.

## Background

Human Papilloma Virus (HPV) is the necessary cause of cervical cancer [1] and several screening methods have been developed to assess its prevalence in risk groups or patients [2] Although 15 HPV types are associated with high risk (HR) for cervical cancer, two HPV types, 16 and 18, account for 70% of cervical cancer cases [3]. Each HR HPV type associates different carcinogenetic risks [4]. Using HPV/DNA genotyping tests can inform on the natural history of HPV infection by type and collectively, determine incidence, prevalence, and progression of HPV infection. Furthermore, screening for a broad array of HPV types can inform decisions on the need for HPV vaccine in a given population and enable early detection of women who are infected with HPV and at risk for cervical cancer.

Romania has the highest incidence of cancer in Europe, with an incidence of 23.9/100,000 [5]. In Romania, systematic screening for cervical cancer (i.e., cytology or HPV/DNA testing) is not available. Furthermore, no data on HPV genotype prevalence in Romania is available [6]. A government attempt in 2009 to implement HPV vaccination in Romanian young girls failed because of the lack of medical information on the prevalence of HPV genotypes.

The goal of this study was to establish HPV prevalence and genotype distribution in women with and without cervical lesions in Northeast Romania (a population of 4.5 million people) in an attempt to gather sufficient information to support future vaccination program.

## Results and discussion

After 2 years of systematic testing the following results were obtained in our study group with regards to classical risk factors for developing cervical cancer: 71/514 (13.8%) of interviewed subjects declared use of oral contraceptives; 146/514 (28.4%) had other gynecologic infections (*Neisseria gonorrhoeae, Candida spp, Trichomonas vaginalis, Treponema pallidum*); 85/514 (16.5%) reported cigarette smoking, while and 53/514 (10.3%) declared having more than three sexual partners. Papanicolau smear test results were as follows: ASCUS - 71/514 (13.8%), ASCH - 49/514 (9.5%), LGSIL - 107/514 (20.8%), HGSIL - 41/514 (8.0%), inflammatory - 31/514 (6.0%) while a normal Pat test was reported for 164/514 (31.9%) patients. Note that 51/514 (9.9%) did not had any cytology examination prior to the study, while presenting with macroscopic and colposcopic suspicion for HPV infection. These results are comparable to those reported in other regions of Romania [7].

The overall testing of the samples in our study gave the following relative prevalence results: out of the total 514 tested samples, 192 (37.3%) samples were positive for HPV/DNA, 120 of which (23.3%) were single HPV type infections and 72 (14%) tested positive for multiple HPV types (Table 1).

**Table 1:** The prevalence of HPV genotypes for all infections

HPV TYPE	Frequency	Percent
NEGATIVE	322	62,6

16	54	10,5

53	28	5,44

51	26	5,05

52	21	4,08

18	15	2,91

31	14	2,73

CP6108	13	2,52

6, 45 *	12	2,33

33	11	2,14

42	10	1,94

58,	9	1,75

68, 73 *	8	1,55

66	7	1,36

84	6	1,16

62, 70*	5	0,97

82	4	0,77

55	3	0,58

56	2	0,38

11, 35, 39, 40, 61, IS39 *	1	0,19

**Figure 1:**
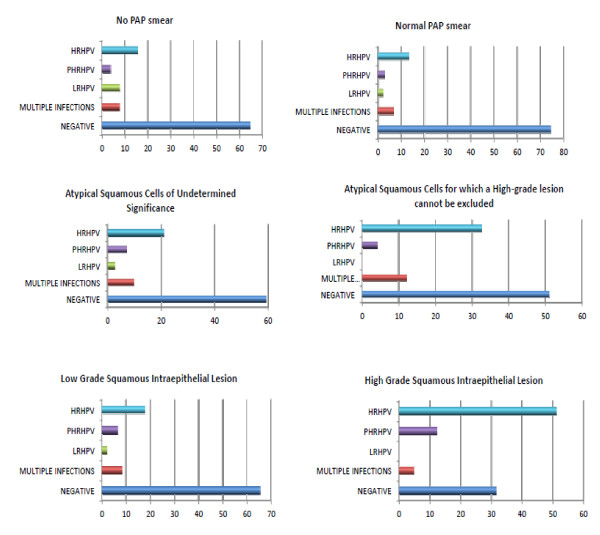
**HPV genotype prevalence in 483 women with/and without cervical lesions from Northeast Romania**. (on the X axis are the percentages for each category according to the classification IARC for HPV types: HRHPV, PHRHPV, LRHPV; total cases: 483 - except 31/514 cases with inflammatory cytology) (HR HPV types: High Risk HPV types, pHR HPV: probably High Risk HPV types, LR HPV types: Low Risk HPV types).

HR HPV types were detected in 8/108 (7.42%) of women with no Pap test and in different percent for patients with Pap smear scores: 15/108 (13.9%) *ASCUS*, 16/108 (14.8%) ASC-H, 19/108 (17.6%) LGSI, 21/108 (19.4%) HGSIL and 7/108 (6.5%) having "inflammatory" cytological result. In 22/108 (20.4%) cases positive for HR HPV, the result of conventional smear was reported as "normal" (Figure 1)

Since the relative prevalence of different HPV type may inform the composition of the polyvalent vaccine in a given region, we focused on the eight first HPV types detected that were HPV 16, 53, 51, 52, 18, 31, CP6108, 6 and HPV 42. The highest prevalence for multiple HPV type infection was observed in the age group of 21-30 years: 19/40 (47.5%), while women in the age group of 31-40 years had the highest prevalence of HR HPV types: 40/108 (37%). Of all the HR HPV positive women detected, 20/71 (18.5%) declared use of oral contraceptives, 32/146 (29.6%) had associated gynecological pathology, 31/85 (28.7%) reported cigarette smoking and 16/53 (14.8%) reported more than 3 sexual partners. Of all the DNA/HPV negative women, 11.3% declared use of oral contraceptives, 27% had associated gynecological pathology, 12.6% reported cigarette smoking and 5.5% reported more than 3 sexual partners.

Multiple HPV types infections were found in 8/72 (11.1%) persons who were never tested for Papanicolau cytology and 20/72 (27.8%) women with normal result. For patients that received other scores, these percents varied between 5.6 and 20.8%. One explanation for detecting DNA/HPV in women with normal cytology is that the majority of HPV infections are transient.

In the group of patients that were tested for cytologic examination, we have detected 18/51 (35.3%) positive with HR HPV (high risk oncogenic types), pHR HPV (probable high risk types), LR HPV (low risk oncogenic types) and multiple HPV type infections [8]

The prevalence of HPV genotypes included in the quadrivalent vaccine was as follows: HPV type 16-10.5% (54/514), HPV type 18-2.91% (15/514), HPV type 6-2.33% (12/514) and HPV type 11-0.19% (1/514).

Women identified to carry with HR HPV infection will follow the European Guidelines for quality assurance in cervical cancer screening (2008): if the cytology is normal, they will repeat the HPV DNA test after 6-12 month; if they present with an abnormal Pap smear, they will be monitored by repetition of the smear, and referral for colposcopy. Women with HR HPV and HGSIL usually are candidate for excisional procedures, but the choice of treatment for women with HSIL will depend on the suspected diagnosis, the size and type of transformation zone, age and fertility aspirations [9].

The relative prevalence of HPV types was similar in patients infected with single genotypes and in those with multiple infections. Multiple infections were produced by two to eight genotypes per sample. Note that these results may be due to either a numerous reexposure rate or to a high sensitivity of the assay used in our study in comparison to other HPV genotyping techniques (*INNO LiPA, Reverse Line Blot) *[10]. In order for the typing to meaningful, quality control and standardized panels of samples would need to be implemented for the regional reference laboratories [11].

Thus, our study identified a high prevalence of HPV infection associated with cervical cancer in our area and therefore supports implementation of primary prevention of cervical cancer by vaccination in Northeast Romania, and in all country as well.

In our region, screening of cervical cancer is not systematic and the quality control guidelines for conventional smears are not implemented [12]. Additionally, general population lack knowledge regarding connection between HPV and cervical cancer and necessity of performing regular screening for detecting precancerous cervical lesions [13]. However, as supported by our study, a systematic testing for HPV both in general population as well as in gynecological patients has to potential to significantly reduce the rates of cervical cancer and therefore a strategy for systematic screening is badly needed.

It is considered that full spectrum genotyping is useful for evaluation HPV epidemiology, and to the study of the impact of HPV vaccination and for clinical practice, to assess the persistence of HPV infection: stratification of risk for CIN 2+, ASCUS follow-up or treatment follow up. However, in Romania, similar to numerous other nations, screening for cervical cancer is not systematic and even conventional smear is not done on a regular basis [12]. In addition, cytology has a lowest sensitivity in comparison with DNA/HPV tests (53% *versus *96%) [14]. This calls for an alternative strategy consisting of improving the infrastructure for molecular diagnostic to enable systematic HPV detection using generic tests [9,15,16] followed by genotyping of the positive samples in regional reference centers. Also, implementation of a systematic screening program in Romania using generic cost effective DNA/HPV detection tests, would further imply an improvement of clinical sensitivity (cross hybridization with HR genotypes) and specificity (i.e., increase the detection cut-off of the assays) of screening tests. Also, DNA/HPV tests should be validated analytically and clinically to be adequate for diagnostic use [16].

There is currently an open national cervical cancer screening pilot study undergoing in Romania. However, this study is still regional and aims to a 10% coverage only. These numbers are clearly different from those observed in other EU countries (i.e., in Netherland the coverage is 77%). This demonstrate that in order to significantly improve the efficacy of cervical cancer screening there is an important need for education of women as well as of medical professionals too [17]. At the same conclusion reached Apostol et al, regarding implementing in Romania of one organized cervical cancer screening [18]. Arbyn M. et all identified in 2007 the burden of cervical cancer in Romania requiring implementation of one well-organized cervical cancer program; the same authors published in 2010, by joinpoint regression method that the incidence and mortality through cervical cancer in Romania is increasing [19,20].

Being a new member of EU, in Romania there are still challenges regarding starting of one organised programmes for cervical cancer: the main reason is the lack of the infrastructure necessary to implement it [21].

Even though our pilot study was carried out on a limited number of patients, it covered a large number of patients and generated results that may permit the policy makers to initiate cervical cancer screening and vaccination programs for teenagers.

## Conclusions

In developing countries as Romania, HPV testing can be used in primary cervical cancer screening. This will be beneficial for patients who will thus avoid excessive referrals to colposcopy and overtreatment [9,14,22].

In the future, screening via HPV/DNA tests and vaccination must become coordinated, with programs providing the most cost-effective cervical cancer prevention strategies.

Our results regarding epidemiological issues are comparable with WHO data at global and European level [6]. Also, they are similar with the overwhelming epidemiological evidence for the role of sexual activity in the transmission of anogenital HPV infections [23,24].

The efficiency of prevention programs (screening, vaccination) is highest if the aim of these programs is focused on community needs. In this respect, the high prevalence of HR HPV documented by our study justifies entirely the necessity of cervical cancer prevention programs in Romania.

## Methods

The study was carried out between September 2009 and November 2011, and included 514/1896 consecutive women (aged: 17-84 years, median: 36.5) from the "Cuza-Vodă" Gynecology Clinical Hospital. All patients were monitored by clinical gynecological examination, cytology exam and some of them, by colposcopic examination.

Women with abnormal Pap smears (ASCUS--*Atypical Squamous Cells of Undetermined Significance*, ASC-H--*Atypical Squamous Cells for which a High-grade lesion cannot be excluded*, LGSIL--*Low Grade Squamous Intraepithelial Lesion*, HGSIL--*High Grade Squamous Intraepithelial Lesion *or inflammatory result) or with only suggestive colposcopy exam were genotyped for HPV infection.

Each participant signed an informed consent and the study was approved by the Bioethical Committee of the "Gr. T. Popa" University of Medicine and Pharmacy of Iaşi, and each woman completed a questionnaire concerning possible cofactors for cervical cancer (e.g., smoking, genital coinfections, oral contraceptive use, number of sex partners), and provided cytological results of previous Papanicolau tests.

Cervical cells were sampled with a cervical brush and they were transported in *Cobas PCR Cell Collection Media *(*Roche*). DNA purification was done using the *High Pure PCR Template *(*Roche*); HPV detection was performed using the *Linear Array HPV Detection *kit (*Roche*), and then DNA was genotyped with the *Linear Array HPV Genotyping Test (Roche, Iaşi, Romania*). Genotyping via this technique involves one step of PCR amplification (*ABI 9700*): the final volume contained 50 μl of purified DNA and 50 μl of master mix (*Roche*). The thermal profile included the next steps: HOLD program: 2 min/50°C; an initial denaturation of 9 min at 95°C was followed by 40 cycles of 95°C for 30 s, 55°C for 1 min, and 72°C for 1 min, with a final extension for 7 min at 72°C. The amplicons have been denatured at the end of PCR amplification.

HPV types were detected by hybridizations of the amplicons. Hybridization was performed in a *TwinCubator *(*HAIN, Life Science*) using Working Hybridization Buffer, Working Ambient Wash Buffer, Working Stringent Wash Buffer, Working Conjugate, Working Citrate Buffer and Working Substrate (*Roche*). The technique was validated through the use of positive and negative controls at each shift; the strips contain two bands for beta globin, which checks if the sample was correctly processed.

*Linear Array HPV Genotyping test *(Roche) is detecting 14 HR HPV (*high risk*) types (16, 18, 31, 33, 35, 39, 45, 51, 52, 56, 58, 59, 68) and 24 pHR HPV (*probably high risk*) and LR HPV (*low risk*) types (6, 11, 26, 39, 40, 42, 45, 53, 54, 55, 61, 62, 64, 66, 67, 69, 70, 71, 72, 73, 81, 82, 83, IS39, CP6108). We validated our HPV genotyping technique by obtaining proficiency for all the HPV types included in WHO HPV Proficiency Study, 2010.

For the main HR HPV types (16, 18, 33, 52) we confirmed the results of LA genotyping result by qPCR/MX3005P (*Stratagene *manufacturers) detection using quantitative evaluation.

## List of abbreviations

HPV: Human papilloma virus; HR HPV types: High Risk HPV types; pHR HPV: Probably high risk HPV types; LR HPV types: Low risk HPV types.

## Competing interests

The authors declare that they have no competing interests.

## Authors' contributions

URG: HPV/DNA purification, PCR amplification, hybridization, results analysis, manuscript preparation. OM and ND: selection of patients, sampling the cervical cell. ILS: director of grant research, searching for founding, study design and manuscript preparation. All authors read and approved the final manuscript.
